# Malignancy validation in a United States registry of rheumatoid arthritis patients

**DOI:** 10.1186/1471-2474-13-85

**Published:** 2012-05-31

**Authors:** Mark C Fisher, Victoria Furer, Marc C Hochberg, Jeffrey D Greenberg, Joel M Kremer, Jeff R Curtis, George Reed, Leslie Harrold, Daniel H Solomon

**Affiliations:** 1 Massachusetts General Hospital, 55 Fruit St., Boston, MA, 02114, USA; 2 Department of Rheumatology, New York University (NYU) Hospital for Joint Diseases, 301 East 17th Street, Suite 1410, New York, NY, 10003, USA; 3 Departments of Medicine and Epidemiology and Preventive Medicine, University of Maryland School of Medicine, 10 S. Pine St., MSTF 8-34, Baltimore, MD, 21201, USA; 4 Department of Rheumatology, New York University (NYU) Hospital for Joint Diseases, 301 East 17th Street, Suite 1410, New York, NY, 10003, USA; 5 Center for Rheumatology, Albany Medical College, State University of New York, 1367 Washington Avenue, Albany, NY, 12206, USA; 6 Department of Medicine, University of Alabama, 510 20th St. South FOT 805D, Birmingham, AL, 35294, USA; 7 Division of Preventive and Behavioral Medicine, University of Massachusetts Medical School, 377 Plantation Street, Worcester, MA, 01605, USA; 8 Department of Medicine, University of Massachusetts Medical School, 377 Plantation Street, Worcester, MA, 01605, USA; 9 Division of Rheumatology, Immunology and Allergy, Brigham and Women’s Hospital, 1620 Tremont Street, Suite 3030, Boston, MA, 02120, USA

**Keywords:** Malignancy, Rheumatoid arthritis, Registry, Validation

## Abstract

**Background:**

Physician reporting is commonly used to ascertain adverse events or outcomes measured in epidemiologic studies. However, little is known on the accuracy of physician reported malignancies compared to pertinent medical record review in large cohort studies.

**Methods:**

The Consortium of Rheumatology Researchers of North America (CORRONA) registry gathers physician-completed questionnaires for rheumatoid arthritis (RA) patients, including request for information on incident malignancies, approximately every three months. For incident malignancies reported from October 1st, 2001, through December 31st, 2007, we retrospectively requested completion of a Targeted Adverse Event (TAE) form for additional information as well as primary source documents to adjudicate the malignancy reports. CORRONA has employed a prospective request for source documentation for these events since 2008. We classified each malignancy as definite, probable, possible, or not a malignancy.

**Results:**

From 20,837 RA patients enrolled in CORRONA, 461 incident malignancies were initially reported on physician questionnaires. After review of returned source documents with adjudication, 234 were deemed definite, 69 probable, 101 possible, and 57 not an incident malignancy. The positive predictive value (PPV) of initial physician report of a malignancy *versus* “definite or probable” malignancy based on adjudication was 0.66 (95% CI 0.61 - 0.70). The PPV was 0.68 (95% CI 0.63 – 0.72) when the subsequent TAE form also confirmed the presence of malignancy. When possible malignancies were included, the PPV of physician-reported malignancies without a subsequent TAE form increased to 0.86 (0.83 – 0.89), and with a subsequent TAE form, 0.89 (0.85-0.91).

**Conclusion:**

Twelve percent of initial physician reports of incident malignancy could not be confirmed with review of source documents. The most common reason for lack of confirmation was inability to obtain documents or insufficient data in source materials. These results suggest that timely collection of relevant medical records and an adjudication process are required to improve the accuracy of cancer reporting in epidemiologic studies.

## Background

Rheumatoid Arthritis (RA) is an auto-immune, multi-system inflammatory disease with significant morbidity and mortality. In addition to its articular manifestations, RA has a variety of extra-articular complications, including malignancy [1,2]. The impact of immunosuppressive treatments used to manage patients with RA may also influence the development of malignancies, as each of these treatments cause perturbations of the immune system that may lead to malignancy [3-6]. Because of the influences of disease activity and therapy on the immune system of patients with RA, as well as the relative infrequent occurrence of many types of malignancy, it can be difficult to assess the impact of a specific medication on the risk of malignancy.

To date, several epidemiologic and pharmacoepidemiology studies have been performed to assess the rate of malignancy in RA patients treated with Tumor Necrosis Factor a inhibitors (TNFi) [7-18]. Sources for these datasets include national registries, prospective cohorts, and administrative databases. Cancer outcomes in these studies have been identified based on national cancer registers, patient self-report, physician report, administrative claims, and medical records. In the United States, common methods for ascertainment of malignancy among RA patients include patient self-report or physician report. Another approach is mining of centralized administrative databases, often using claims data or ICD-9 codes to document presence of malignancy, as in the US, national malignancy registries do not exist. The Surveillance Epidemiology and End Results (SEER) registry does capture data on incident malignancies in the US, but it only collects data for a handful of states as well as some additional, separate urban areas. There are also state specific cancer registries, however, between the overall number of RA patients and the overall low rate of malignancy among RA patients, they often do not have the power necessary for pharmacoepidemiologic studies. In addition, individual patient information is not available in many of these registries to permit linkage to observational registries of other diseases. Outside the United States, in addition to the above methods, many countries have national cancer registries where reporting of all incident malignancies to a central database with confirmation is mandatory.

To classify incident cancers in a large cohort of patients with RA where a cancer registry was not available, primary records were requested to validate the malignancy. We developed an adjudication process and tested the accuracy of physician-reported incident cancers, using pertinent medical records as the gold standard.

## Methods

### Study population

The Consortium of Rheumatology Researchers of North America (CORRONA) is an independent registry of RA patients that has collected clinical, laboratory, imaging, medication, and toxicity data since 2001[19]. To date, it has collected data from over 100 rheumatology practices and over 300 participating rheumatologists throughout the United States, both academic and private, with over 20,000 RA patients enrolled. Data are collected from both patients and their treating rheumatologists using questionnaires, which gather information on disease duration, prognostic information, physician and patient-determined standardized disease severity and activity measures, medical comorbidities, use of medications including DMARDs, laboratory values, and adverse events [20]. Follow-up assessments are requested at four month intervals and completed during routine clinical encounters. Approvals for participation in the CORRONA registry are obtained from the respective Institutional Review Boards of participating academic sites and a central Institutional Review Board for private practice sites.

At each visit, physicians complete questionnaires that include information about new comorbidities, including cancer. For the period of this study (10/01 to 12/07), follow-up Targeted Adverse (TAE) Forms and request for source documents were performed retrospectively. Since 2008, TAE forms and requests for source documents are initiated at the time of the initial physician report. Only the reporting rheumatologists’ office can request medical records due to privacy requirements established by the CORRONA registry. The medical records requested include pathology reports, hospital discharge summaries, notes from an oncologist, and/or notes from a primary care physician.

### Source documents

After the adverse event forms were returned, all sites were asked to provide source documents to corroborate the diagnosis of an incident malignancy. If source documents were not received within a specified period of follow-up, a second request was made. If source documents were still not submitted, a second questionnaire asking for validation specifically of the organ of the malignancy, pathology report, and date of onset was requested.

### Medical record review

All malignancies reported between 10/1/2001 and 12/31/2007 were evaluated for this analysis. Once all data, including the adverse event forms and primary source documents, were received, they were reviewed using a structured abstraction form. In the analyses reported here, all data were reviewed and abstracted separately by two investigators (MF and VF). The abstracted data were then compared, and inconsistencies were resolved by returning to the source documents and assessing which record was accurate. As some data were subject to interpretation, any discrepancies between the two records were noted, and a third party (JG) reviewed the data. Three investigators (MF, VF, JG) then discussed the information. If consensus between MF and VF could not be reached, the third party (JG) adjudicated how the data would be recorded.

Source documents were ranked by two of the authors (MF and VF) for confidence in confirmation of the presence and type of malignancy. These rankings were then submitted to other members of the research team (DHS, JG, JK, GR, MH), and a final hierarchy of the level of confidence in the cancer diagnosis was constructed. The hierarchy gave greater weight to objective evidence of malignancy (pathology report) or note from the appropriate type of expert (e.g. oncologist, radiation oncologist, dermatologist for skin cancers, etc.). Other sources included death certificates, admission notes, and discharge summaries ( 1: Table S1).

### Adjudication of malignancies

**Figure 1:**
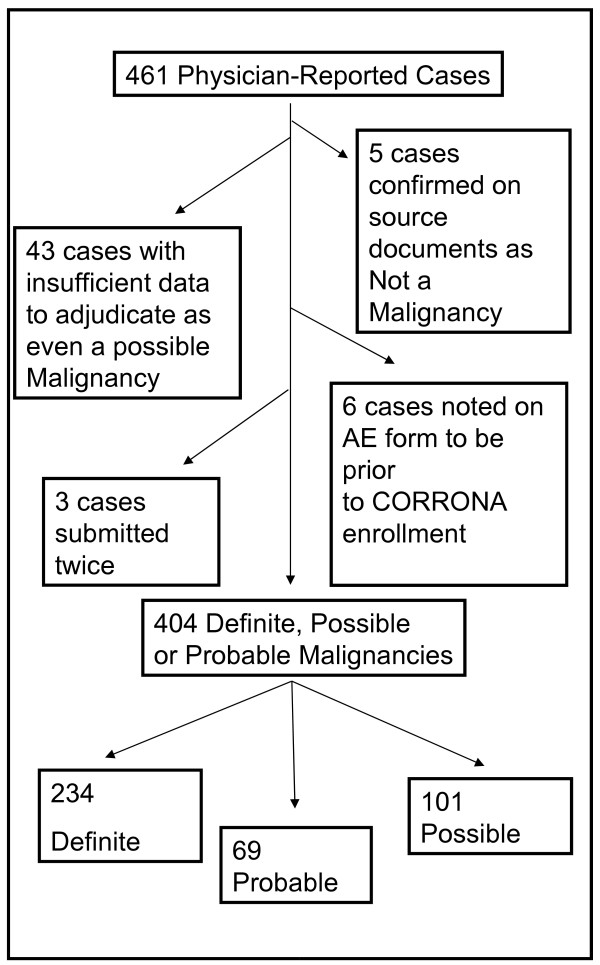
Disposition of physician reported cases*.* This represents the results of the adjudication of the 461 reported malignancies in 401 patients

Source documents were reviewed for each malignancy; the document with the highest hierarchical value was recorded (see above). Malignancies were defined as “definite” if a malignancy was reported on a biopsy report or from an oncologist or radiation oncologist. To be considered an incident malignancy, the date of diagnosis (month and year) also had to be submitted, and had to have occurred after enrollment in CORRONA. For certain malignancies, note from an appropriate specialty was considered sufficient to designate the case as “definite” (ex: skin cancer and dermatologist’s note). If the supporting documentation was a note from another type of specialist, and the date of diagnosis and histology were included either in the note or hand written on adverse event form, then the diagnosis was designated “probable”. If either the histology or the date of diagnosis were not present, then the case was designated “possible.” In some cases, the source documents or adverse event form noted that the case was not a malignancy. Disposition of reported cases is shown in Figure 1.

### Statistical analysis

Outcomes for this analysis compared a gold standard (documentation of confirmed incident malignancies) to the CORRONA questionnaire as well as the adverse event form report of malignancy. Positive predictive value (PPV) of follow-up visit report and adverse event form was calculated compared to the gold standard with 95% confidence intervals (CI).

## Results

The demographics of patients in this analysis are shown in Table 1. Of the 20,839 RA patients in the CORRONA Registry, 401 had at least one rheumatologist-reported malignancy during follow up, and some patients had more than one incident malignancy reported during follow up. RA patients with malignancies differed from the subjects without malignancy: they were older (65.2 *vs.* 57.6 years, p < 0.0001), less likely to be female (63 *vs.* 73%, p <0.0001), and were more likely to be non-Hispanic White (89 *vs.* 84%, p = 0.0005). In addition, they also had longer disease duration at enrollment (12.6 *vs.* 9.6 years, p < 0.0001) and longer duration of follow-up within CORRONA (2.3 *vs.* 1.8 years, p < 0.0001). There was no significant difference in seropositive status between the two groups (71 *vs.* 68%, p = 0.31).

**Table 1:** Patient Demographics

	Total CORRONA Population	Total CORRONA Patients Without Malignancy	Total CORRONA Patients With Malignancies	P-Values
	(N = 20,839)	(N = 20,438)	(N = 401)**	
Age (Mean, SD)	57.7 (13.6)	57.6 (13.9)	65.2 (11.0)	<.0001
Female Gender (N, %)	15,135 (72.8%)	14,883 (73.0%)	252 (62.8%)	<.0001
Race/Ethnicity (N, %)				
White	17,292 (84.0%)	16,940 (84.0%)	352 (88.7%)	0.0005
Hispanic	1,291 (6.3%)	1,275 (6.3%)	16 (4.0%)	
Black	1,248 (6.1%)	1,238 (6.1%)	10 (2.5%)	
Asian	344 (1.7%)	340 (1.7%)	4 (1.0%)	
Other	418 (2.0%)	403 (2.0%)	15 (3.8%)	
Seropositivity (N, %)	7,400 (67.7%)	7,224 (67.6%)	176 (70.7%)	0.31
RA Disease Duration at Enrollment in CORRONA (mean years, SD)	9.56 (9.8)	9.49 (9.8)	12.56 (11.3)	<.0001
Years of Follow-up After CORRONA Enrollment Until Report of Cancer* (mean, SD)	1.76 (1.6)	1.75 (1.8)	2.26 (1.7)	<.0001

* follow-up time censored at the time of the first report of an incident malignancy.

** 44 patients had multiple malignancies reported, for a total of 461 reported malignancies.

The various documents used to determine confidence in the diagnosis of malignancy are shown in Table 2. Of the 461 cases, there were corroborating documents for 303 (65.7%). For cases with multiple documents, the document with the highest validity as deemed by the authors was used. Source documents that were able to definitely confirm the diagnosis were available for about half of the reported malignancies. For 158 cases (34.3% of the reported malignancies), there was no additional confirmation beyond an adverse event form. Of these, 36 (22.8% without confirmation, 7.8% overall) were adjudicated as probable, as the AE form submitted both a histology and an incident date. An additional 79 (50% without confirmation, 17.1% overall) were adjudicated as possible incident malignancy. The remaining 43 (27.2% without confirmation, 9.3% overall) had insufficient data on the TAE Form and they were deemed unlikely to be an incident malignancy.

**Table 2:** Hierarchy of Source Documents

Type of Document	Level of Confidence*	N, % Used**
Biopsy report	1	173 (57.1%)
Oncologist Note	1	58 (19.1%)
Radiation Oncologist Note	1	9 (2.3%)
Dermatologist note (skin cancers only)	1	6 (2%)
OB-GYN Note (for OB-GYN cancers only)	1	1 (0.3%)
Urologist Note (for GU cancers only)	1	2 (0.7%)
Admission note	2	2 (0.7%)
Discharge summary	2	5 (1.7%)
Rheumatologist Note	2	16 (5.3%)
Other Physician Note	2	28 (9.2%)
Pharmaceutical Company adverse event form	3	3 (1.0%)

* For Level 1, sufficient independently to confirm a diagnosis of malignancy

For level 2, if a date of diagnosis (month and year) on the adverse event form or in the note was also provided, and a histology was submitted, defined as a Probable malignancy. If either of that data was absent, defined as a Possible malignancy.

For level 3, if tissue diagnosis included, defined as a Probable malignancy. If not, defined as a Possible malignancy.

** For cases with multiple source documents, only the document with the highest hierarchical value was used. 303 cases had submitted documents (Total N).

Reasons why cases were excluded (N = 57) from categorization as incident malignancies are shown in Figure 1. The most common reason for exclusion was determining, usually *via* source documents, that there was insufficient data to determine whether an incident malignancy had occurred (43 cases, 75.4% of excluded, 9.3% overall). Six cases were excluded as they were prevalent, but not incident malignancies (10.5% of excluded, 1.3% overall). Five cases were excluded as on corroboration, they were confirmed to not be malignancies (8.8% of excluded, 1.1% overall). Lastly, 3 cases were excluded as they were submitted twice (5.3% of excluded, 0.7% overall). All 234 definite malignancies had corroborating data to confirm date of onset and histology. Of the 69 probable malignancies, 36 had no corroborating records submitted, but had an incident date and history on the TAE form. Of the 101 possible malignancies, 79 had no records submitted. Of the possible malignancies, 60 had a histology submitted but no date of the malignancy. 13 had a date of the malignancy but no histology, and 28 had both no histology and no date.

The number and type of malignancies stratified by adjudicated status are found in Table 3. The most common reported malignancies were non-melanoma skin cancers (NMSC), 154 total cases, comprising 33.4% of all malignancies. Using the gold standard of medical records review, just under half (46.1%) of these reported NMSC could be classified as definite malignancies; adding probable cases increased that number to 68.8%. In addition, there were 9 melanomas reported, one of which was ultimately confirmed not a melanoma. The second most common reported malignancy was breast cancer (59 cases, 13% of all reported malignancies). Five different histologies were noted on records review (data not shown). About three-quarters (74.6%) of reported breast cases were adjudicated as definite or probable. Hematologic, prostate, lung, and colon cancers all accounted for between 4.6% - and 9.1% of the total reported malignancies. Lastly, 57 cases (11.1%) were proven on either adverse event forms or from source documents to actually not be an independent incident malignancy. This was most common with cancers that were not able to be specified (71.9% of all cases deemed not an incident malignancy). These cases were included in the analysis as on the TAE they were confirmed as malignancies by the primary site. In one case, a histology and incident date was listed but the primary organ was listed as “unknown.” This case was deemed a definite incident malignancy.

**Table 3:** Reported Incident Malignancy Types by Adjudication

Organ or Type of Malignancy	Physician Form	Records Review – Definite*	Records Review – Probable**	Records Review – Possible***	Records Review – Not Malignancy or Not An Incident Malignancy****
	(Total: 461)	(Total: 234)	(Total: 69)	(Total: 101)	(Total: 57)
Skin – NMSC^1^	154	71 (46.1%)	35 (22.7%)	42 (27.3%)	6 (3.9%)
Skin – Melanoma	9	5 (55.6%)	1 (11.1%)	2 (22.2%)	1 (11.1%)
Skin – Other^2^	5	4 (80%)	1 (20%)	0	0
Breast	59	41 (69.5%)	3 (5.1%)	13 (22.0%)	2 (3.4%)
Hematologic^4^	42	32 (76.2%)	7 (16.7%)	3 (7.1%)	0
Other^3^	49	1 (2.0%)	0	7 (14.3%)	41 (83.7%)
Prostate	38	21 (55.3%)	4 (10.5%)	11 (29%)	2 (5.3%)
Lung	25	11 (44.0%)	2 (8%)	9 (36%)	3 (12%)
Colon^5^	21	13 (61.9%)	5 (23.8%)	3 (14.3%)	0
Thyroid	9	3 (33.3%)	5 (55.5%)	1 (11.1%)	0
Bladder	12	6 (50%)	2 (16.7%)	3 (25%)	1 (8.3%)
Head and Neck^6^	7	4 (57.1%)	2 (28.6%)	1 (14.3%)	0
Uterine	5	3 (60%)	0	1 (20%)	1 (20%)
Upper GI^7^	4	4 (100%)	0	0	0
Renal	4	2 (50%)	1 (25%)	1 (25%)	0
Cervical	3	1 (33.3%)	0	2 (66.7%)	0
Ovarian	3	2 (66.7%)	0	1 (33.3%)	0
Pancreatic	2	2 (100%)	0	0	0
Bile duct	1	1 (100%)	0	0	0
Parotid Gland	2	1 (50%)	0	1 (50%)	0
Brain	2	1 (50%)	1 (50%)	0	0
Hepatic	2	2 (100%)	0	0	0
Peritoneum	1	1 (100%)	0	0	0
Gallbladder	1	1 (100%)	0	0	0
Angiosarcoma	1	1 (100%)	0	0	0

1) Includes 47 squamous cell, 91 basal cell, and 16 unspecified.

2) Includes vulvar, anal, perianal, intergluteal skin cancers.

3) Includes all malignancies with unspecified primary organ.

4) Includes 36 lymphomas, 1 Myeloma, 3 Leukemia, 2 Myelodysplasia.

5) Includes Rectal, anal, bowel, small intestine, appendix.

6) Includes Larynx, tongue, vocal cord, nasopharyngeal.

7) Includes esophageal and gastric.

* Definite malignancy defined as date of diagnosis and histology confirmed on records from an oncologist, biopsy, radiation oncologist, or appropriate specialist for specific cancers.

** Probable malignancy defined as date of diagnosis and histology confirmed from records other than oncologist, biopsy, radiation oncologist, or appropriate specialist.

*** Possible malignancy defined as submission of records confirming either date of

diagnosis or histology but not both.

**** Not Malignancy or Not An Incident Malignancy defined as records confirming pathology were benign or date of diagnosis prior to enrollment in CORRONA

A comparison of the predictive value of the CORRONA questionnaire and the adverse event form to the gold standard of medical records review is shown in Table 4. Using only definite and probable cases, the questionnaire had a positive predictive value (PPV) of 0.66 (95% CI 0.61 – 0.70). If possible cases were added, this increased to 0.86 (95% CI 0.83 – 0.89). If information from the TAE form was also used, the PPV was 0.68 (95% CI 0.63 – 0.72) for definite and probable cases. If possible cases were included, the PPV increased to 0.89 (95% CI 0.85 – 0.91).

**Table 4:** **Predictive Values*versus*Gold Standard of Records Review**

	PPV Physician Questionnaire (95% CI)	PPV Adverse Event Form (95% CI)
	0.66	0.68
Definite/Probable	(0.61–0.70)	(0.63–0.72)
	0.86	0.89
Definite/Probable/Possible	(0.83–0.89)	(0.85–0.91)

## Discussion

Accurate classification of incident malignancies is essential for pharmacoepidemiologic studies, as well as to compare rates between different cohort studies and assess the relationship between disease characteristics and treatments with the development of cancer. Patient self-report may be an imperfect method for ascertaining incident malignancies, as the difference between a benign mass and true neoplasm is not always clearly communicated or understood. Even physician report may be unreliable, as the treating rheumatologist may not have the primary information about an incident malignancy. Administrative databases based on claims data are also not ideal, as the methodology used to define malignancy can cause the positive predictive value to vary widely [21,22]. We attempted to validate physician report of incident malignancy based on physician questionnaires and adverse event forms using a gold standard of pertinent medical records review in a large cohort of patients with RA. We were able to confirm approximately two-thirds of reported incident malignancies. We found that having a positive report of incident malignancy on the adverse event forms increased the PPV only slightly compared to questionnaires alone. However, the PPV of the physician report alone may not be sufficiently high enough to be relied on for epidemiologic purposes. Hence, use of non-validated reports of incident malignancy might overestimate the true incidence in patients with RA and might bias the results of pharmacoepidemiologic studies assessing causal associations with specific treatments.

Our second notable finding was the exclusion of 14 malignancies (3.0%) because they were either duplicate entries, established cancers that were not incident, or proven by corroborating documents to not be malignant. An additional 43 cases (9.3%) had insufficient data and could not even be deemed possible malignancies. Clearly some of those are incident cases, although it is not clear how many. In addition, 101 cases (21.9%) could at best be classified as possible incident malignancies (either histology report or date of diagnosis missing). The accuracy of classifying cancer in a study assessing the risk of different treatments is important. If the possible malignancies are dismissed as non-cancers, it might artificially create a sense of decreased risk for a given medication, when at least some of those cases are likely to be true malignancies. Alternatively, including all possible malignancies might change the perception of the risk/benefit ratio and prevent patients or physicians from choosing a medication that could have benefit. The first approach would provide greater specificity; however, this is at the sacrifice of sensitivity. Assessing both rates would provide a range within which the true value most likely exists, but also illustrates the degree of uncertainty generated by physician report and even follow-up adverse event forms.

Our third significant finding was that despite follow-up with the primary treating rheumatologist, almost a third of the time, we could not obtain source documents to corroborate the malignancy. In some cases, this may be due to lack of diligence by the site. However, in most cases, this was because records truly could not be obtained. As a result, since 2008 the registry now requests source documents at the time the cancer is initially reported, to avoid requesting records of events from multiple years earlier. This proactive approach has improved the proportion of cases with source documents.

The methodology defines the classification of the malignancies, as it does depend on appropriate documentation submitted from the patient’s rheumatologist. The use of appropriate specialists for specific malignancies, such as a dermatologist for a skin cancer or a urologist for a genitourinary cancer, was deemed appropriate as for those malignancies where that specialist will treat the patient directly, as opposed to having an oncologist manage treatment. All ‘possible’ malignancies were classified as such because of some inadequacy of the requisite data to have confidence that it was truly an incident case.

The type of dataset itself also can impact the rate of malignancy found in a given population. The intensity of evaluation is much higher in a clinical trial, and virtually all cases of malignancy during the follow-up period would be expected to be reported. However, Phase II and III randomized controlled trials only follow patients with a single intervention for a finite period, and thus cannot offer the kind of data that can be possible in a long-term disease registry. It is possible that the intensity of surveillance itself can impact clinical care and outcome (Hawthorne effect), but it is still unclear if it will affect the overall incidence of a specific comorbidity such as malignancy in patients with RA.

The strengths of this analysis include the use of a very large dataset which includes information gathered from both providers and patients in the context of an established infrastructure which permitted the identification and follow-up of all reported cases. This allowed us to procure source documents in most cases. In addition, we used a standardized record review process with multiple adjudicators. All of these steps increased the validity of the results.

This study did have certain weaknesses as well. Although standard questions on the development of new malignancies were included in both the physician and patient questionnaires at each visit, it is still possible that some malignancies were not reported. As previously noted, the absence of a national cancer registry in the US makes independent assessment of new malignancies extremely difficult if not impossible. As such, we cannot calculate a negative predictive value of the default evaluation of “no malignancy.” Individual state cancer registries are available, are sponsored by both the NCI and the CDC, and their reliability continues to improve, though they are not reflective of nationwide rates, as the SEER data is. However, identification of individual patients requires the use of personal health information (PHI), and the CORRONA consent does not permit the use of PHI to cross-link with other registries. In addition, despite repeated attempts to obtain source documents, in many cases none were available. This led to the classification of many malignancies as “possible,” even while reported independently on the primary questionnaire from either the patient or treating physician. Certainly many of these cases likely were true malignancies. We believe that this problem is likely to be endemic to all observational registry studies in the absence of a national cancer registry. The response rate for pertinent records did improve when cases were reported to CORRONA more recently. However, there was no significant difference in rate of excluded cases (either not an incident malignancy or confirmed to be not a malignancy) from earlier *versus* more recent cases. A cancer registry where all malignancies are reported and validated, as is frequently done outside the United States, is superior to our methodology. However, information from within the United States is still of great value, as drug utilization patterns in the US are quite different from European registries where penetration of biologic agents is significantly greater than in Europe[23,24]. We therefore believe that it is critical to appropriately analyze data on major comorbidites from a US source.

## Conclusions

In conclusion, our process of confirming malignancies started with the identification of possible cases reported at the time of a routine clinic visit. It was further refined through subsequent hierarchical steps which included a targeted adverse event form and subsequent review and ranking of available source documents by a team of physicians. As a result of these rigorous adjudication steps, we found that routine reports of malignancy by patients and physicians were not always accurate. The implications of over and underreporting in large disease registry may be epidemiologically significant. We believe that it would be ideal if uniform standards for reporting of these events could be adopted in observational disease registries where cancer registries are not available.

## Competing interests

Dr. Fisher has nothing to disclose. Dr. Furer has nothing to disclose. Dr. Hochberg has consulted for Abbott Laboratories, Amgen, Bristol Myers Squibb, Genentech, UCB Inc. Dr. Greenberg receives salary support from research grants from the NIH (K23AR054412), the Arthritis Foundation and the National Arthritis Research Foundation, serves as Chief Scientific Officer for CORRONA and has served on Advisory Boards for Genentech and Pfizer. Dr. Kremer is the President of CORRONA, receives salary support. Dr. Curtis has consulted for or received honoraria from Roche/Genentech, UCB, Centecor, CORRONA, Amgen, and Pfizer, and has received research support from Amgen, Roche/Genentech, Centecor and CORRONA. Dr. Curtis also receives salary support from the NIH (AR053351). Dr. Harrold received support from the NIH (K23AR053856). She also has consulted for CORRONA. Dr. Solomon has received research support from Amgen and Abbott for rheumatoid arthritis research and has received support for an educational course from Bristol Myers Squibb. Dr. Solomon also receives salary support from the NIH for mentoring (K24 AR 055989).

## Authors’ contributions

MCF, VF, JD, and DH participated in the study design, data acquisition, data analysis, and manuscript preparation. MCH, JK, JC, GR, and LRH participated in data analysis and manuscript preparation. All authors read and approved the final manuscript.

## Pre-publication history

The pre-publication history for this paper can be accessed here:

http://www.biomedcentral.com/1471-2474/13/85/prepub

## Supplementary Material

Additional file 1 **Table S1.** Rank of Source Documents. Click here for file
