# Supplementary material for: Malignancy validation in a United States registry of rheumatoid arthritis patients
Source: BMC Musculoskelet Disord. 2012 May 31;13:85. doi: 10.1186/1471-2474-13-85 (PMC3403943; doi:10.1186/1471-2474-13-85)
Supplement: Additional file 1 — Table S1. Rank of Source Documents. [file 1471-2474-13-85-S1.doc]

Supplemental Table 1: Rank of Source Documents

| Type of Document | Hierarchical Rank |
| --- | --- |
| Biopsy | 1 |
| Oncologist Note | 2 |
| Radiation Oncologist Note | 3 |
| Dermatologist note (skin cancers only) | 4a |
| OB-GYN Note (for OB-GYN cancers only) | 4b |
| Urologist Note (for GU cancers only) | 4c |
| Admission note | 5 |
| Discharge summary | 6 |
| Rheumatologist Note | 7 |
| Other Physician Note | 8 |
| CORRONA AE form only | 9 |
| Pharmaceutical Company AE form | 10 |
